# Increased secreted PLA2 in epithelial cells promotes the progression of chronic non-atrophic gastritis to chronic atrophic gastritis through the TGF-β signaling

**DOI:** 10.1371/journal.pone.0343531

**Published:** 2026-03-04

**Authors:** Hairong Hao, Yanjun An, Yuan Liu, Baole Li, Ran Zhang, Yingli Hao, Yuanyuan Pan, Na Li, Junfeng Kang

**Affiliations:** 1 China Academy of Chinese Medical Sciences, Xiyuan Hospital, Shanxi Hospital, Taiyuan, Shanxi, China; 2 Affiliated Hospital of Shanxi University of Traditional Chinese Medicine, Taiyuan, Shanxi, China; 3 The First Clinical College of Shanxi University of Traditional Chinese Medicine, Taiyuan, Shanxi, China; Universidade de Trás-os-Montes e Alto Douro: Universidade de Tras-os-Montes e Alto Douro, PORTUGAL

## Abstract

**Introduction:**

The progression of Chronic gastritis seems to follow a pattern from chronic non-atrophic gastritis (CNAG) to chronic atrophic gastritis (CAG) to cancer, so it is particularly important to block key targets in disease progression. A gene that synthesizes secreted phospholipase A2, attracted our attention.

**Objectives:**

To study whether phospholipase A2 group 10 (PLA2G10) in epithelial cells promote the progression of CNAG to CAG through the transforming growth factor-β (TGF-β) signaling.

**Methods:**

We used RNA microarray and single-cell RNA sequencing datasets for bioinformatics analysis. The effects of PLA2G10 were verified by *in vivo* and *in vitro* experiments. The *in vivo* experiments used SD rats to establish a CNAG model for PLA2G10 and TGF-β intervention to observe the effects on gastric mucosal inflammation. *In vitro* experiments were conducted using human gastric mucosal epithelial cells (GES-1) for similar interventions.

**Results:**

PLA2G10 inhibition led to the downregulation of TGF-β expression and attenuated the inflammatory response of the gastric mucosa. And the blockade of TGF-β signalling delayed the progression of CNAG to CAG, as evidenced by a reduction in inflammatory cell infiltration, a more regular cellular arrangement, and a reduction in collagen deposition.

**Conclusions:**

Our study shows that PLA2G10 plays a key role in the progression of chronic gastritis and highlights the important role played by the TGF-β signalling pathway in this process.

## 1 Introduction

Chronic gastritis (CG) refers to a persistent inflammatory reaction of the gastric mucosa, and comes to one of the most common diseases seen in endoscopy, usually manifests as epigastric pain, bloating, indigestion, nausea, vomiting, belching, loss of appetite, and other symptoms [1,2]. According to the Sydney system [3] for the classification of CG, it can be categorized into chronic non-atrophic gastritis (CNAG) and chronic atrophic gastritis (CAG). It has been estimated the prevalence of CAG to be approximately 15% in the US population [4]. In another large-scale cross-sectional survey, among the 8892 patients diagnosed with CG, 17.7% were CAG and 82.3% were CNAG [5]. *Helicobacter pylori* infection is the main cause of CG, other common causes include bile reflux, long-term use of drugs such as non-steroidal anti-inflammatory drugs, alcohol consumption, and autoimmune factors. Accordingly, primary purpose of CG treatment is to eliminate the underlying cause, improve gastric mucosal inflammation, relieve symptoms, and prevent complications. Guidelines at all levels generally recommend individualized treatment based on etiology.

In terms of histopathology, inflammatory cell infiltration of gastric mucosa is the common feature of CNAG and CAG, but CAG is also accompanied by the typical features of atrophy, intestinal metaplasia and dysplasia, which are commonly known as gastric precancerous lesions, and are considered to be crucial steps in the progression from gastritis to gastric cancer [6,7]. On the other hand, CNAG and CAG, as two types of gastritis, are different from each other, but also related. This sequence of pathological changes can be described as the slow formation of CNAG, then with atrophic progression at a rate of 3% per year, to the appearance of specialized intestinal type epithelium, and then to atypical hyperplasia/intraepithelial neoplasia [8]. Therefore, CNAG can be regarded as a more serious CAG, and exploring the pathological mechanism of CNAG progression to CAG and blocking it pharmacologically is a potential direction for CG therapy.

The persistent inflammatory reaction in the gastric mucosa is recognized as an initial driver for the development of CNAG to CAG [9]. Along with the infiltration of immune cells such as lymphocytes, plasma cells and macrophages caused by stimulation, local resident epithelial cells and fibroblasts in gastric mucosal tissues respond accordingly [9,10]. It has been reported changes in the types and quantities of these immune cells within CG are closely intertwined with the disease development and prognosis [11]. Notably, the response of these locally resident cells is often profound, varies of genes, including cytokines, immune-related receptors and signaling pathways, may be induced and play a role in the ongoing progression of inflammation [12,13]. Benefit from the rapid rise of gene sequencing technology, more and more genes involved in CG development have been excavated [14,15]. However, due to the huge volume of genomic results, study sponsors are often unable to make the most thorough description of the experimental results, nor can they fully verify the obtained experimental results. Therefore, in-depth reanalysis and verification of sequencing results are still valuable.

Bioinformatics is a new discipline that collects and analyzes genetic data, it takes genomic DNA sequence information analysis as its source, which has been widely used in the mining and exploration of CG-related hub genes [16,17]. It is not only a reanalysis of the published results of the database, but also a comprehensive analysis of the research results of different research sponsors around a certain proposition. Therefore, in order to better understand the key pathological mechanisms of CNAG to CAG development at the genetic level, our approach involved three main steps. Firstly, we conducted a comprehensive RNA microarrays dataset analysis of differential gene expression in CG dynamic progression using the GSE116312 dataset, and phospholipase A2 group 10 (PLA2G10), a gene that synthesizes secreted phospholipase A2, attracted our attention. Subsequently, we cross-referenced this dataset with the GSE134520, a single-cell RNA sequencing dataset encompassing both CNAG and CAG, resulting in PLA2 expression changes occurred primarily in pit mucous cells (PMCs), a secretory subtype of gastric mucosal epithelial cells, and may promote the progression of CNAG to CAG through transforming growth factor-β (TGF-β) signaling. Finally, we use molecular biology method to verify the above hypothesis.

## 2 Materials and methods

### 2.1 Experimental protocol

**Fig 1:**
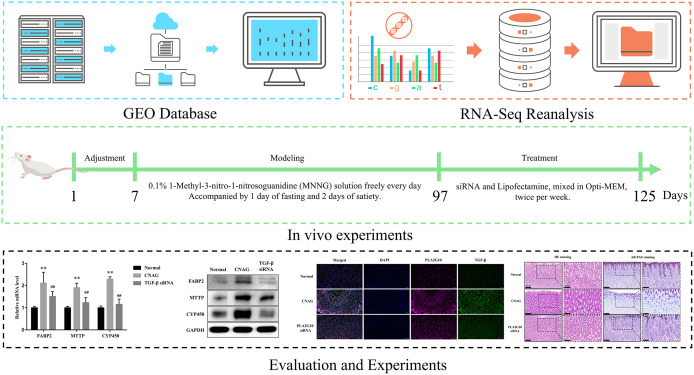
Experimental protocol. Notes: In general, this study comprises four parts. **(A)**. Accessing databases and performing data collection. **(B)**. Data re-analysis. **(C)**. Designing in vivo and in vitro experiments based on the data re-analysis results. **(D)**. Sample detection and data analysis validate the in vivo findings.

In general, this study comprises four parts (Fig 1): Part 1. We accessed the NCBI Gene Expression Omnibus dataset GSE116312 (RNA microarrays) and analyzed gastric biopsy RNA microarray data from seven CNAG patients and three CAG patients. Part 2. We conducted an analysis of single-cell RNA-seq data (GSE134520) from gastric biopsies of three CNAG patients and three CAG patients, and compared the results with those from Part 1. Part 3. We designed *in vivo* and *in vitro* models using PLA2G10 siRNA and TGF‑β siRNA to investigate their upstream–downstream relationship and their effects on the pathological progression of CNAG. Part 4. We performed data detection and statistical analysis on the experimental samples by using pathological sections, immunofluorescence, and quantitative assessment of relative gene and protein expression levels.

### 2.2 Data acquisition

We have used two datasets for data mining of publicly available data present on NCBI gene expression omnibus: GSE116312 of RNA microarrays and GSE134520 of single-cell RNA sequencing, respectively. We acquired RNA microarrays data of seven follicular gastritis patients and three CAG patients, single-cell RNA sequencing data of three CNAG patients and three CAG patients. As mentioned in the original publications, all the studies requiring the clinical sample were approved by Institutional Review Board.

### 2.3 RNA microarrays data analysis

The main process of RNA microarrays data analysis is differentially expressed genes (DEGs) identification and enrichment analysis [18]. Preprocessing steps for the data included log_2_ conversion and normalization. The data (GSE116312) were analyzed using the R software packages “limma” to find DEGs in two groups, *P* < 0.05 and |log_2_ Fold Change| > 1 were regarded as significant. Then, using the R software packages, “ggplot2” and “pheatmap” to create the principal component analysis (PCA) maps, volcano plots, and heat maps. The Gene Ontology (GO) enrichment analysis is a common method for examining biological processes (BP), cellular components (CC), and molecular functions (MF). Following the division of DEGs into two groups, including the up DEGs and the down DEGs. DEG gene ontology analyses were conducted using the “cluster Profiler” package of R software.

### 2.4 Single-cell RNA-sequencing data analysis

All algorithm used for analysis of single cell RNA seq data (GSE134520) was based on Seurat，R toolkit for single cell genomics with custom modification. The combined raw data of CNAG and CAG samples obtained from the gene expression omnibus were read on RStudio and Seurat object for the same has been created. Individual cells with less than 200 or more than 2000 unique gene count and more than 30% of reads arising from mitochondrial genes were filtered out. Under these conditions, we obtained 28,397 single cells for downstream analysis. PCA was used for dimensional reduction based on the top 3000 most variable genes. Variable genes were determined using the Find Variable Genes function of Seurat. The Find Clusters function of Seurat was used on PCA reduced genes (dims = 1:30). Harmony method [19] was operated for data integration. Harmony iteratively adjusts the PCA coordinates of cells to minimize batch effects between different samples while preserving biological differences. The Harmony algorithm iterates several times to adjust cell positions until batch effects are sufficiently removed. Uniform manifold approximation and projection (UMAP) was used for visualization of clusters on a 2-D map. DEGs were determined by the Find Markers function of Seurat, which is based on the non-parametric Wilcoxon Rank sum test. Annotating each cluster based on known cell type marker genes. PLA2G10 was detected in all cells using Feature Plot. To quantify the enrichment of cell clusters across disease conditions, we compared the observed and expected cell numbers in each cluster by computing the Ro/e value using the “Epitools” R package according to the following formula: Ro/e = observed/ expected, in which Ro/e is the ratio of observed cell number over the expected cell number of a given combination of cell cluster and tissue. The expected cell number for each combination of T cell clusters and tissues are obtained from the chi-squared test. GO-BP enrichment analysis was also operated to analyze across different cell types, comparing enrichment under two conditions.

### 2.5 In vivo experiment design

Thirty-two 2-month-old SD male rats, 170–210 g, purchased from Jiangsu Jicui Pharmachem Biotechnology Co. were used. Animals were housed in a specific pathogen-free, laminar-flow housing apparatus under 25 ± 2°C temperature, 55 ± 5% humidity, 12 h light/dark regimen, and maintained on standard rodent pellet diet. All animal experimental protocols were approved by the Medical Ethics Committee of Shanxi University of Traditional Chinese Medicine (AWE202209054). All experiments were conducted in accordance with the National Institutes of Health Guidelines for the Care and Use of Laboratory Animals.

Rats were randomly divided into four groups: Normal, CNAG, PLA2G10 siRNA, TGF-β siRNA. After one week of adaptive feeding, except for rats in Normal group, CNAG model was constructed in other groups according to Wang J described previously [20]. Briefly, rats were fed 0.1% 1-Methyl-3-nitro-1-nitrosoguanidine (MNNG) solution freely in sterile drinking water, protected from light, and replaced every 48 hours. Exposure was continuous for 90 days, and CNAG model were successfully established, then *in vivo* RNA interference began. PLA2G10 siRNA and TGF-β siRNA groups received gavage intervention performed as previously described by Park [21] et al. with some modifications. Briefly, 5 nmol siRNA were combined with 0.25 mL of lipofectamine 2000 in 0.5mL of Opti-MEM for gavage, twice a week for four weeks. Meanwhile, the other groups received gavage with empty vectors carrying non‑targeting siRNA sequences, supplied by the commercial siRNA kit, at the same frequency, serving as controls. Rats were euthanized by intraperitoneal injection of 3% pentobarbital sodium salt solution at a dose greater than 100 mg/kg, depending on body weight.

### 2.6 Sample collection

When all interventions had concluded, i.e., when the rats had reached 7 months of age, they were euthanized via intraperitoneal injection of 3% pentobarbital sodium solution at a dose exceeding 100 mg/kg, adjusted for body weight. Gastric tissue from each rat was dissected on ice‑cold glass plates. After the gastric contents were rinsed with phosphate‑buffered saline, the stomach was rapidly divided on ice into two parts along the limiting ridge separating the forestomach (non‑glandular) from the glandular stomach. The entire procedure was completed within 2 minutes, and the glandular mucosa was preserved. One portion of the glandular mucosa was fixed in 10% neutral‑buffered formalin for histological examination, and the remaining portions were stored at −80 °C for subsequent quantitative analyses.

### 2.7 In vitro experiment design

GES-1 human gastric mucosal epithelial cells were purchased from Immocell Co. and cultured in DMEM supplemented with 10% fetal calf serum under a humidified 95% air and 5% CO_2_ atmosphere. Passages 3−6 of the GES-1 were used for the experiments. It is consistent with the experimental design *in vivo*, GES-1 were divided into four groups: Normal, CNAG, PLA2G10 siRNA, and TGF-β siRNA. CNAG model was constructed by 20 μM MNNG challenge for 8 h [22], and RNA interference was performed by 20 pmol siRNA combined with 1 μL of lipofectamine 2000 in 2 μL mL of Opti-MEM.

### 2.8 Histological analysis

Gastric tissues were fixed with 10% neutral buffered formalin, and embedded in paraffin, cut into sectioned 5-μm thick slices. HE and Alcian Blue-Periodic Acid Schiff (AB-PAS) staining were carried out according to the instructions of the stain kit, respectively. Sections were observed under a Leica DMI3000B microscope, with the use of bright field.

### 2.9 Immunofluorescence

Briefly, paraffin section was dehydrated, and autofluorescence quenching agent was added, then blocked with Block Buffer at room temperature. The primary antibody and the fluorescent secondary antibody were added successively. DAPI was added dropwise and the nuclei were stained. Carl Zeiss LSM 800 laser confocal inverted fluorescence microscope was used to observe and capture at the corresponding excitation wavelength.

### 2.10 Quantitative real-time PCR (qPCR)

Total RNA from gastric tissues or GES‑1 cells was extracted using the TRIzol method, and the RNA OD values were subsequently measured. The reverse transcription of RNA was performed using Prime Script RT reagent Kit. Primer was designed and synthesized by Shanghai Biotechnology Service Company in accordance with Gene sequence in GenBank Gene sequence design, together with Oligo v6.6 (Sequences as S1 Table). qPCR was performed using Premix Ex Taq SYBR-Green PCR (Takara) according to the manufacturer’s instructions on an ABI PRISM 7300 (Applied Biosystems, Foster City, CA, USA). The mRNA level of individual genes was normalized to GAPDH and calculated by the 2^−ΔΔCT^data analysis method.

### 2.11 Western blotting (WB)

Total protein of gastric tissues or GES-1 were obtained by adding RIPA lysate, grinding and homogenizing, centrifuging at 12,000 rpm for 10 min, and the protein concentration were measured in accordance with the instructions for the preparation of the BSA standard curve. Then, according to the determination of protein concentration and the volume of the sample on the sample, electrophoresis, membrane transfer, BSA closed, add the corresponding primary antibody (1:1000), incubated with secondary antibody, and exposed.

### 2.12 statistical analysis

Animals or data points were not excluded and each experiment was repeated two times, and data were presented as mean ± standard deviation (SD). Statistical analysis was performed using GraphPad Prism 8.0 Software (San Diego, CA, USA). Group comparisons were assessed with Student’s t-test or one-way ANOVA for comparison of multiple columns. A value of *P* < 0.05(two-tailed) was considered as statistically significant.

## 3 Results

### 3.1 RNA microarrays data revealed the progression of CNAG to CAG was related to PLA2G10

**Fig 2:**
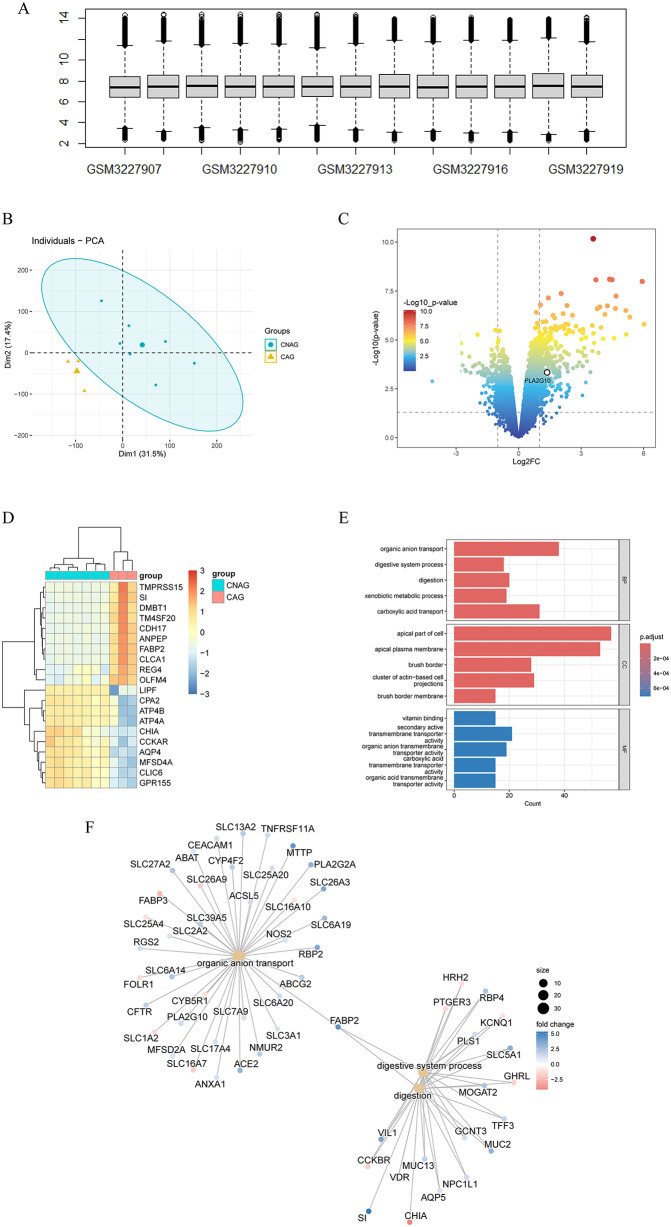
RNA microarrays data revealed the progression of CNAG to CAG was related to PLA2G10. Notes: **(A)**. The box diagram reflected the sequencing depth of each tissue sample. **(B)**. Individual-PCA plot of gene expression in CNAG (cyan) and CAG samples (croci) revealed strong clustering of samples by phenotype. **(C)**. Volcano plot representation of DEGs analysis in CNAG and CAG. **(D)**. DEGs heat map of CNAG and CAG. **(E)**. Bar chart showed the results of GO analysis conducted by DEGs of CNAG and CAG. **(F)**. Network-based prioritization of DEGs in “organic anion transport” pathway and “digestion”.

To gain a better understanding of the key genes involved in the progression of CNAG to CAG, we acquainted GSE116312 which included transcriptomic landscape of seven CNAG patients and three CAG patients. The box diagram (Fig 2A) showed that all samples had consistent sequencing depth, and individual-PCA (Fig 2B) revealed strong clustering of samples by phenotype. Besides, CAG resulted in the up-regulation of 331 genes and down-regulation of 129 genes compared with CNAG (Fig 2C), and the most significant genes included the up-regulation of *TMPRSS15*, *SI*, *DMBT1*, *TM4SF20*, *CDH17* while the down-regulation of *LIPF*, *CPA2*, *ATP4B*, *ATP4A*, *CHIA* and other genes (Fig 2D). GO enrichment analysis (Fig 2E) showed that CAG significantly affected BP successively organic anion transport, digestive system process, digestion, xenobiotic metabolic process, carboxylic acid transport, and the analysis results of CC and MF were consistent with BP. It is not difficult to understand that the evolution of CNAG to CAG involves biological functions such as digestion, metabolism, and acid transport, but the enrichment of DEGs in organic ions has aroused our interest. Therefore, we extract and classify the DEGs enriched in this BP, the results showed the (Fig 2F) up-regulation of inflammation-related genes *TNFRSF11A* and angiotensin converting enzyme 2 (ACE2), the up-regulation of CAG function-related genes fatty acid binding protein 2 (FABP2), microsomal triglyceride transfer protein (MTTP) and cytochrome P450 4F2 (CYP4F2), while the expression of *SLC* family, as the main carrier of the organic anion transport function, were irregular among subfamilies. In addition, combined with literature studies [23,24], we determined that highly expressed PLA2G10 may be the common upstream of genes related to inflammation and CG function, suggesting that it may play a key role in the progression of CNAG to CAG.

### 3.2 PLA2G10 secreted by gastric epithelial cells may promote the progression of CNAG via TGF-β signaling

**Fig 3:**
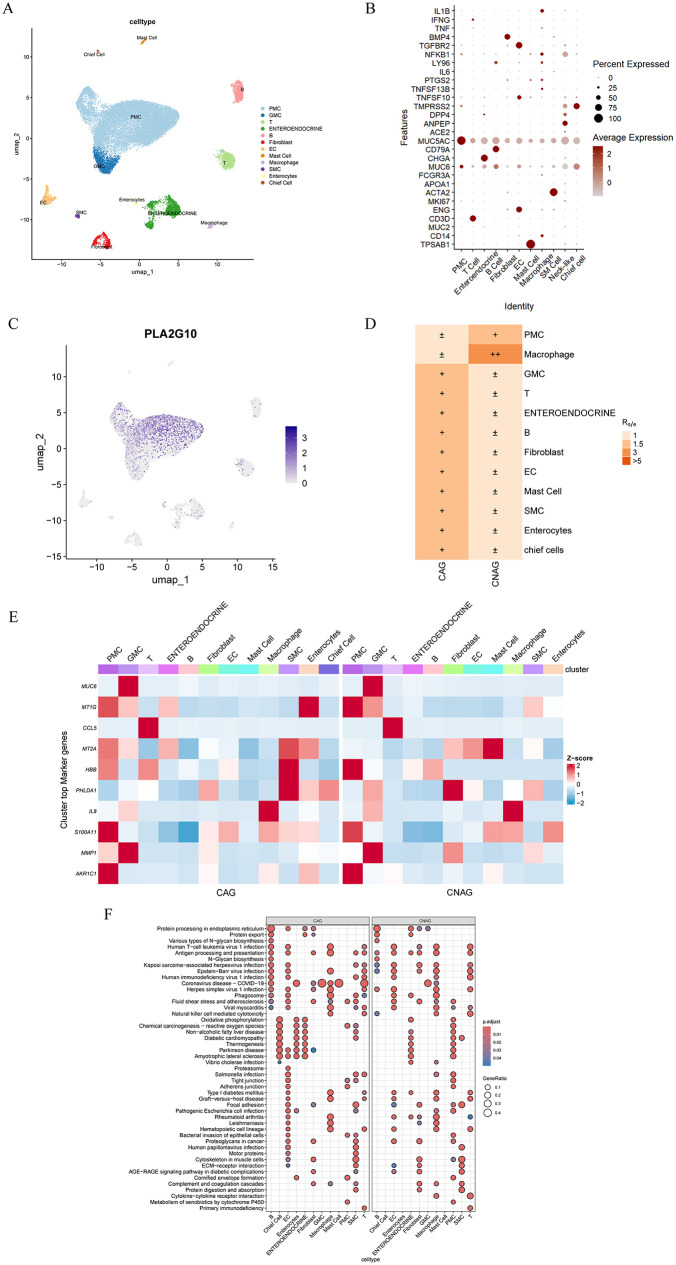
PLA2G10 secreted by gastric epithelial cells may promote the progression of CNAG via TGF-β signaling. Notes: (A). UMAP dimensionality reduction results of single-cell RNA sequencing data. (B). Dot plot illustrated the expression of various genes across different cell types. (C). UMAP plot showed the expression of the gene PLA2G10 in single-cell RNA sequencing data, indicated by the color. (D). CAG and CNAG condition preferences of human gastric cell types revealed by Ro/e. (E). Marker genes were shown in CNAG and CAG respectively. (F). GO-BP enrichment analysis across different cell types, comparing enrichment under CNAG and CAG.

To further determine which gastric mucosal cell types highly express PLA2G10, we accessed GSE134520, a single-cell RNA-sequencing dataset comprising three CNAG patients and three CAG patients, and analyzed 28,397 single cells derived from gastric mucosal biopsies from these volunteers. After quality control, normalization, PCA, graph-based clustering and UMAP (Fig 3A), we annotated clusters using canonical marker transcripts and a published gastric atlas [25]. Epithelial subsets (Fig 3B) comprised pit mucous cells (PMC; *MUC5AC*, *MUC6*), enteroendocrine cells (*CHGA*, *MUC5AC*), chief cells (*TMPRSS2*) and non-epithelial compartments (Fig 3B) included fibroblasts (*BMP4*), smooth muscle cells (SM Cell; *ACTA2*), endothelial cells (EC; *ENG, TGFBR2*) and immune cells (T cells: *CD3D*; B cells: *CD79A*; mast cells: *TPSAB1*; macrophages: *CD14, NFKB1*).

Based on the cell annotation results, PLA2G10 expression was most pronounced in PMC (Fig 3C), and the proportion of PMC was higher in CNAG than in CAG (Fig 3D). Further, we investigated the DEGs of PMC between CNAG and CAG (Fig 3E), notably, matrix metalloproteinase1 (MMP1) was significantly upregulated in the PMC of CAG. In addition, GO-BP enrichment analysis (Fig 3F) across different cell types under CNAG and CAG showed significant difference in “oxidative phosphorylation”, “ECM-receptor interaction”. It is well known that the transmission of TGF-β signals requires the participation of phosphorylated Smads, which causes the high expression of MMPs downstream to regulate the matrix environment and closely related to inflammation and fibrosis [26,27]. Combined with previous reports of PLA2G10/TGF-β accelerating pulmonary fibrosis [28], we speculated PLA2G10 secreted by gastric epithelial cells may promote the CNAG to CAG via TGF-β signaling.

### 3.3 Inhibition of PLA2G10 down-regulated TGF-β and alleviated gastric mucosal inflammation in vivo

**Fig 4:**
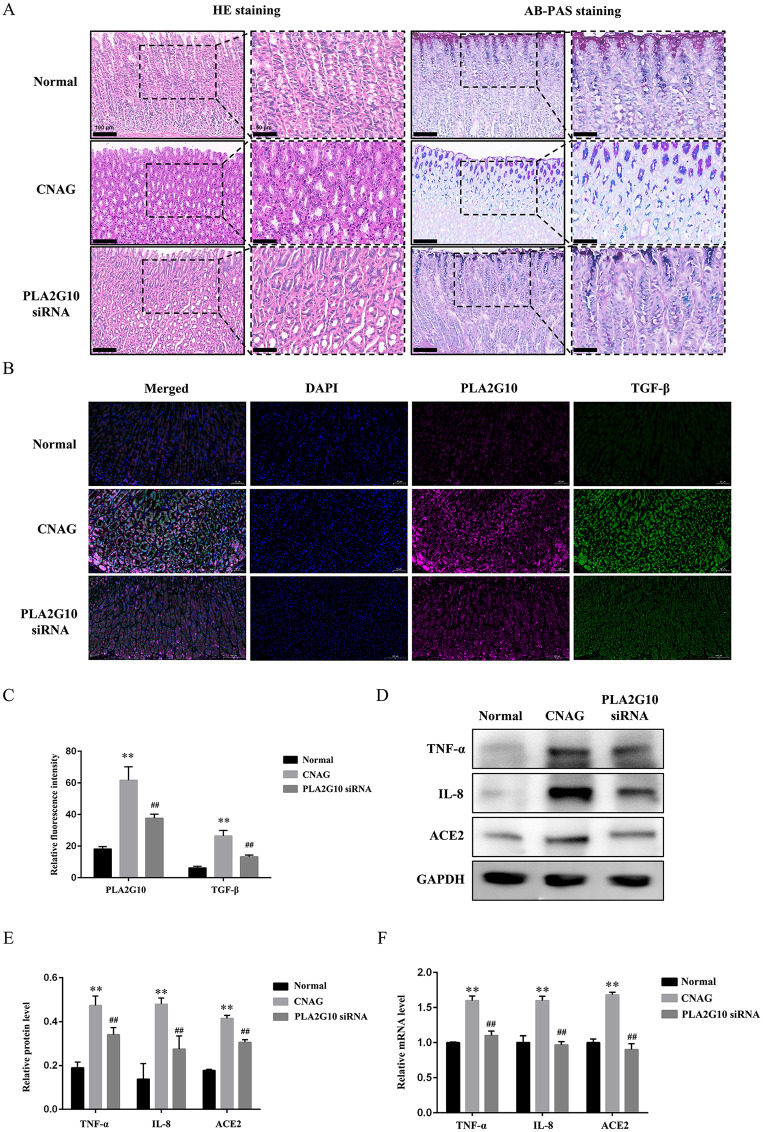
Inhibition of PLA2G10 down-regulated TGF-β and alleviated gastric mucosal inflammation *in vivo.* Notes: (A). Representative HE and AB-PAS staining of gastric mucosa in each group, left side, 200 × , scale bar = 100 μm, right side, 400 × , scale bar = 50 μm. (B). Representative immunofluorescence of gastric mucosa in each group, 400 × , scale bar = 50 μm. (C). Relative fluorescence intensity of PLA2G10 and TGF-β. ^**^P < 0.01 vs. the Normal groups, ^##^*P* < 0.01 vs. the CNAG. (D). Typical WB protein bands of TNF-α, IL-8 and ACE2. (E). Relative protein level of TNF-α, IL-8 and ACE2 in gastric mucosa tissues. ^**^P < 0.01 vs. the Normal groups, ^##^*P* < 0.01 vs. the CNAG. (F). Relative gene expression of TNF-α, IL-8 and ACE2 in gastric mucosa tissues. ^**^P < 0.01 vs. the Normal groups, ^##^*P* < 0.01 vs. the CNAG.

To verify the roles of PLA2G10 and TGF-β signaling in CNAG and to elucidate their relationship, we first established a rat CNAG model using an MNNG solution. Ten days after model induction, rats in the CNAG group exhibited a statistically significant reduction in body weight compared with the Normal group (*P* < 0.01), and two weeks of PLA2G10 inhibition led to a significant increase in body weight in CNAG rats relative to CNAG (*P* < 0.01, S2 Table). HE staining (Fig 4A) showed the gastric mucosa of rats in the Normal group was covered by a single layer of columnar epithelium of uniform size and morphology, arranged neatly. The cytoplasm contained a small number of mucin granules, with no interspersed goblet cells. The lamina propria was filled with densely packed glands, and chronic inflammatory cell infiltration was rare. But in the CNAG group, the gastric mucosa was thinned, the distance between glands increased, and the number of intrinsic glands was reduced. There was stromal hyperplasia between glands and architectural disarray. Locally, there was extensive infiltration by inflammatory cells, mainly plasma cells and lymphocytes, with occasional eosinophils. Vacuolar degeneration was observed in individual glands, and dilatation of glandular lumina was present within the mucosa. In AB-PAS staining, no obvious blue-stained foci of gastric intestinal metaplasia were seen in the mucosal layer of the Normal group, whereas the CNAG group showed diffuse gastric intestinal metaplasia. Inhibition of PLA2G10 reduced both the extent and severity of gastric intestinal metaplasia. Besides, HE and AB-PAS staining were performed on eight rats per group, and pathological changes in the gastric mucosa were quantified. In the Normal group, all eight rats had normal gastric mucosa, with a CNAG incidence of 0%. In the CNAG group, one rat had normal mucosa, five had inflammation, and two had intestinal metaplasia, giving a CNAG incidence of 87.5%. In the PLA2G10 siRNA group, four rats had normal mucosa, three had inflammations, and one had intestinal metaplasia, with a CNAG incidence of 50%. Furthermore, immunofluorescence (Fig 4B) showed the co-localization of PLA2G10 and TGF-β in gastric mucosal tissue, and the fluorescence intensity (Fig 4C) of them were both increased in CNAG model rats compared with the Normal (*P* < 0.01). The intervention of PLA2G10 siRNA significantly reduced the fluorescence intensity of TGF-β (*P* < 0.01), suggesting an inhibition on the expression of TGF-β. In addition, we analyzed both the mRNA and protein level of tumor necrosis factor-α (TNF-α), interleukin-8 (IL-8) and ACE2 (Fig 4D-4F), which were co-enriched with PLA2G10 in our biogenic analysis and shown to be closely related to the inflammatory progression of CNAG. These substances were upregulated in CNAG models than Normal (*P <* 0.01), and downregulated in PLA2G10 siRNA group compared with the CNAG group (*P* < 0.01), suggesting the inhibition of PLA2G10 down-regulated TGF-β and alleviated gastric mucosal inflammation *in vivo*.

### 3.4 Inhibition of PLA2G10 down-regulated TGF-β and alleviated gastric mucosal inflammation in vitro

**Fig 5:**
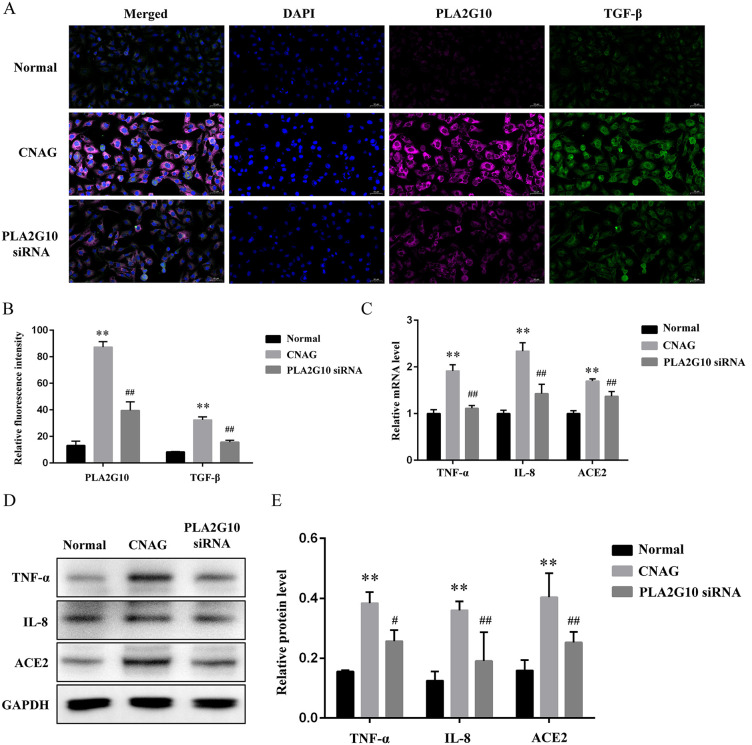
Inhibition of PLA2G10 down-regulated TGF-β and alleviated gastric mucosal inflammation *in vitro.* Notes: (A). Representative immunofluorescence of GES-1 in each group, 400 × , scale bar = 50 μm. (B). Relative fluorescence intensity of PLA2G10 and TGF-β. ^**^P < 0.01 vs. the Normal groups, ^##^*P* < 0.01 vs. the CNAG. (C). Relative gene expression of TNF-α, IL-8 and ACE2 in GES-1. ^**^P < 0.01 vs. the Normal groups, ^##^*P* < 0.01 vs. the CNAG. (D). Typical WB protein bands of TNF-α, IL-8 and ACE2. (E). Relative protein level of TNF-α, IL-8 and ACE2 in GES-1. ^**^P < 0.01 vs. the Normal groups, ^#^*P* < 0.05, ^##^*P* < 0.01 vs. the CNAG.

Next, we established an *in vitro* model of CNAG using human gastric mucosal epithelial cells GES-1 and investigated the effects of inhibiting PLA2G10 on TGF-β and the inflammatory environment. Consistent with the results of *in vivo*, fluorescence intensity (Fig 5A, 5B) of PLA2G10 and TGF-β were both increased in CNAG group compared with the Normal (*P* < 0.01), and the intervention of PLA2G10 siRNA significantly reduced the fluorescence intensity of TGF-β (*P* < 0.01). Besides, both the mRNA (Fig 5C) and the protein level of TNF-α, IL-8, and ACE2 (Fig 5D, 5E) in GES-1, were all upregulated in CNAG than Normal (*P <* 0.01), downregulated in PLA2G10 siRNA group compared with the CNAG group (*P* < 0.05), suggesting the inhibition of PLA2G10 down-regulated TGF-β and alleviated gastric mucosal inflammation *in vitro*.

### 3.5 Blocking TGF-β signal delayed the progression of CNAG to CAG in vivo

**Fig 6:**
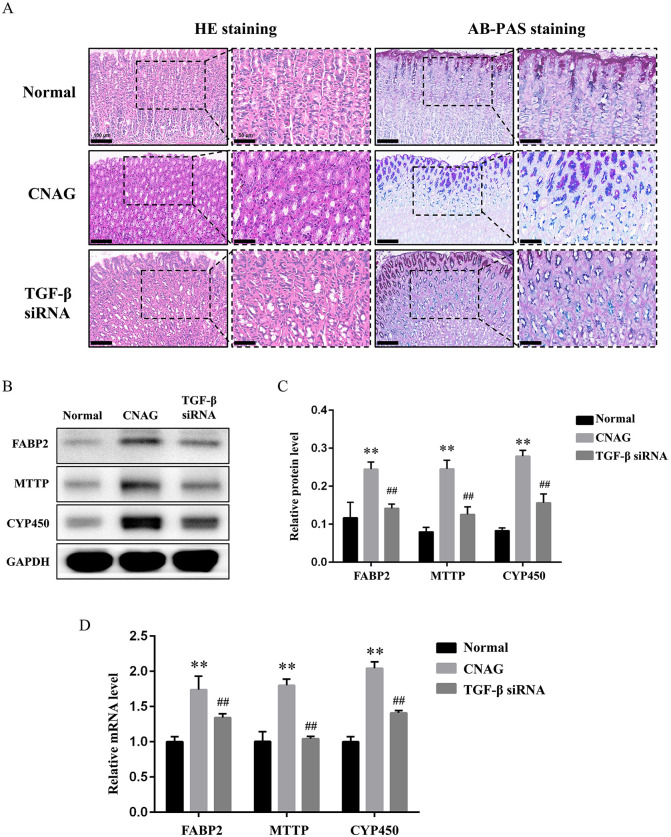
Blocking TGF-β signal delayed the progression of CNAG to CAG*in vivo*. Notes: (A). Representative HE and AB-PAS staining of gastric mucosa in each group, left side, 200 × , scale bar = 100 μm, right side, 400 × , scale bar = 50 μm. (B). Typical WB protein bands of FABP2, MTTP and CYP450 in each group. (C). Relative protein level of FABP2, MTTP and CYP450 in gastric mucosa tissues. ^**^P < 0.01 vs. the Normal groups, ^##^*P* < 0.01 vs. the CNAG. (D). Relative gene expression of FABP2, MTTP and CYP450 in gastric mucosa tissues. ^**^P < 0.01 vs. the Normal groups, ^##^*P* < 0.01 vs. the CNAG.

Made one step further, we investigated the role of TGF-β in the progression of CNAG to CAG. *In vivo*, CNAG rats exhibited increased inflammatory cell infiltration in the gastric mucosa with irregular distribution, accompanied by a degree of intestinal metaplasia. TGF-β siRNA intervention attenuated the pathological progression from CNAG to CAG, evidenced by reduced inflammatory cell infiltration, a more regular tissue architecture, and less intestinal metaplasia (Fig 6A). In the TGF-β siRNA group, three rats had normal mucosa, three had inflammations, and two had intestinal metaplasia, with a CNAG incidence of 62.5%. Subsequently, we examined both gene and protein expression of CG-related factors including detected CG function-related genes FABP2, MTTP and CYP450 (Fig 6B-6D). These factors were upregulated in CNAG models than Normal (*P <* 0.01), and downregulated in TGF-β siRNA group compared with the CNAG group (*P* < 0.01), suggesting the block of TGF-β signal delayed the progression of CNAG to CAG *in vivo*.

### 3.6 Blocking TGF-β signal delayed the progression of CNAG to CAG in vitro

**Fig 7:**
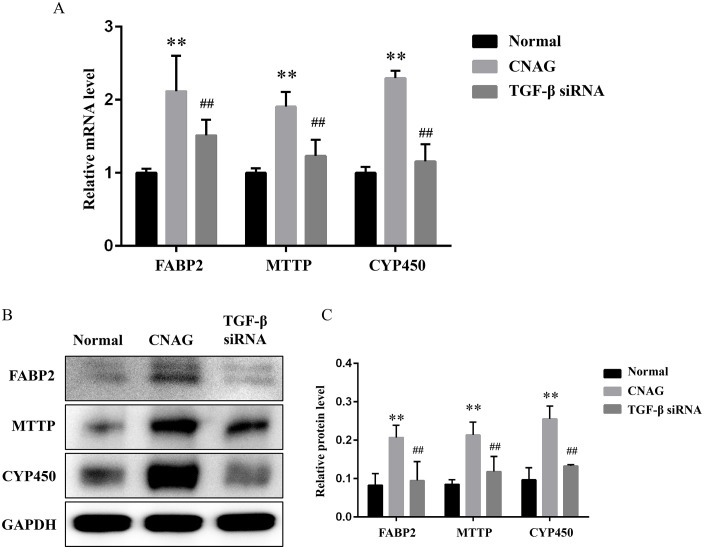
Blocking TGF-β signal delayed the progression of CNAG to CAG *in vitro*. Notes: (A). Relative gene expression of FABP2, MTTP and CYP450 in GES-1 cells. ^**^P < 0.01 vs. the Normal groups, ^##^*P* < 0.01 vs. the CNAG. (B). Typical WB protein bands of FABP2, MTTP and CYP450 in each group. (C). Relative protein level of FABP2, MTTP and CYP450 in GES-1 cells. ^**^P < 0.01 vs. the Normal groups, ^##^*P* < 0.01 vs. the CNAG.

Finally, we investigated whether blocking TGF-β signal could delayed the progression of CNAG to CAG *in vitro*. In consistent with the results *in vivo* (Fig 7A-7C), TGF-β siRNA intervention downregulated both the gene and protein expression of FABP2, MTTP and CYP450 compared with the CNAG group (*P <* 0.01). This also made us more convinced that TGF signaling plays a driving role in the CNAG progression.

## 4 Discussion

In this study, we acquainted data of RNA microarrays and single-cell RNA sequencing to gain a better understanding of the key genes involved in the progression of CNAG to CAG. Bioinformatics analysis revealed that PLA2G10, which is highly expressed in CNAG epithelial cells, may promote disease progression to CAG through TGF-β signal. In the subsequent molecular biology studies, the inhibition of PLA2G10 down-regulated TGF-β and alleviated gastric mucosal inflammation. It was mainly manifested in that the intervention of PLA2G10 siRNA reduced inflammatory cell infiltration in CNAG gastric mucosa tissues, and the cell arrangement was more orderly. Meanwhile, the fluorescence intensity of TGF-β was decreased, and the relative expression levels of genes and proteins of inflammatory markers TNF-α, IL-8 and ACE2 were decreased. The above conclusions are supported by *in vivo* and *in vitro* experiments. On the other hand, TGF-β blocking delayed the progression of CNAG to CAG in animal and cellular studies, manifested in the use of TGF siRNA reduced gastric mucosal inflammation, intestinal metaplasia, and inhibited the relative expression of FABP2, MTTP and CYP450, which were closely related to CG progression.

PLA2G10 is a phospholipase and can excise the SN-2 acyl chain of phospholipids to release free fatty acid and Lys-phospholipid molecules [29]. In contrast to intracellular PLA2G10, secreted PLA2G10 are ideally positioned to cleave phospholipids available on the cell surface or in the extracellular milieu [30]. PLA2G10 secreted from small intestinal Paneth cells, is involved in the shaping of the intestinal microbiota, thereby indirectly affecting cancer and psoriasis in distal skin [31]. Increased PLA2G10 in certain key epithelial cells in the lungs of idiopathic pulmonary fibrosis patients were closely related to the fibrotic and inflammatory properties, it may work through TGF-β signaling [28]. Besides, modulation of immunity by PLA2G10 had also been extensively studied in recent years [32]. In our study, related to the phospholipase activity of PLA2G10, we found the regulatory effect of PLA2G10 secreted by gastric mucosal epithelial cells on TGF-β. Inflammatory-related TNF-α, IL-8 and ACE2 were also regulated by PLA2G10, and they were all confirmed to be positively associated with the progression of CG in clinical studies [33].

On the other hand, FABP2 is a key protein in lipid transport and specifically expressed in the small intestine, traffic lipids from the intestinal lumen to enterocytes and bind superfluous fatty acids to maintain a steady pool of fatty acids in the epithelium. As a lipid chaperone, FABP2 can also carry lipophilic substance to facilitate targeted transport [34,35]. To our knowledge, in this paper, we found for the first time that FABP2 in gastric mucosal tissue is differentially expressed in CNAG and CAG populations, and may act as a downstream of TGF-β to drive CG progression. We hypothesize that when the integrity of the gastric epithelium is compromised, more FABP2 would be released into the circulation, further affected the metabolic transport of stomach.

Similarly, MTTP is an endoplasmic reticulum resident protein that is essential for the assembly and secretion of triglyceride-rich, apolipoprotein B-containing lipoproteins [36]. Mutant mice with conditional intestine-specific deletion of the MTTP gene manifested a significant accumulation of neutral lipids in the villus of the small intestine and a breakdown of the epithelial barrier [37]. Most importantly, Wang N, et al [38] revealed MTTP as a potential biomarker for prognosis and immune cell infiltration in gastric cancer, this was consistent with the use of MTTP as a key indicator of CNAG progression to CAG in our study. The third indicator we chosen was CYP4F2 [39], a gene encodes a member of the cytochrome P450 superfamily of enzymes, and the gene polymorphism of CYP450 has long been closely related to CG progression [40,41].

Bioinformatics analyses provide independent support for our results. At the beginning of the study, we conducted bioinformatics research. GSE116312, which included transcriptomic landscape of seven CNAG patients and three CAG patients, revealed 331 up-regulated genes and 129 down-regulated genes in the progression of CNAG to CAG. The up-regulated DEGs were chiefly enriched in the organic anion transport pathway, including inflammation-related genes TNFRSF11A, ACE2, and CG function-related genes FABP2, MTTP and CYP4F2, and used PLA2G10 as the common regulatory upstream. Given the diversity of cell populations involved in the course of CG, we next acquainted single-cell RNA sequencing data of three CNAG patients and three CAG patients. We observed that the expression of PLA2G10 was most significant in PMC cells, a gastric mucosal epithelial cell with secretory as the main function. Besides, MMP1 was significantly upregulated in the PMC of CAG and GO-BP enrichment analysis across different cell types under CNAG and CAG showed significant difference in “oxidative phosphorylation”, “ECM-receptor interaction”. Based on the extensive regulatory effects of TGF-β on the ECM environment, we hypothesize that PLA2G10 highly expressed in epithelial cells accelerates the transformation of CNAG to CAG via TGF-β signaling.

Our study has several limitations. First, due to limited experience, we did not obtain gross anatomical images of the stomach during tissue collection in the animal experiments, which reduced the macroscopic context of the study. Second, our examination of CG function‑related genes, FABP2, MTTP, and CYP4F2, was confined to phenotypic observations, without in‑depth mechanistic exploration. Investigating the downstream signaling of these pathways will be a key direction for our future work.

In summary，we found evidence of increased PLA2G10 expressed in epithelial cells by bioinformatics method, and proved PLA2G10 could accelerate the transformation of CNAG to CAG via TGF-β signaling. Our study provides new insights into the pathological mechanisms of CG progression, as well as new directions for pharmacological blocking of the disease.

## 5 Conclusions

In conclusion, our study unveils increased secreted phospholipase A2 in epithelial cells promotes the progression of chronic non-atrophic gastritis to chronic atrophic gastritis through the TGF-β signaling.

## Supporting information

S1 Table Nucleotide sequences of primers used for RT-PCR amplification. (DOCX)

S2 Table Rat body weight at different time points. (DOCX)

## Abbrevitions

CG: chronic gastritis
CNAG: chronic non-atrophic gastritis
CAG: chronic atrophic gastritis
PLA2G10: phospholipase A2 group 10
TGF-β: transforming growth factor-β
ACE2: angiotensin converting enzyme 2
CYP4F2: cytochrome P450 4F2
FABP2: fatty acid binding protein 2
MTTP: microsomal triglyceride transfer protein
ECM: extracellular matrix

## References

[1] Sipponen P, Maaroos H-I. Chronic gastritis. Scand J Gastroenterol. 2015;50(6):657–67. doi: 10.3109/00365521.2015.1019918 25901896 PMC4673514

[2] Chinese Society of Gastroenterology, Cancer Collaboration Group of Chinese Society of Gastroenterology, Chinese Medical Association. Guidelines for diagnosis and treatment of chronic gastritis in China (2022, Shanghai). J Dig Dis. 2023;24(3):150–80. doi: 10.1111/1751-2980.13193 37245073

[3] Sipponen P, Price AB. The Sydney System for classification of gastritis 20 years ago. J Gastroenterol Hepatol. 2011;26 Suppl 1:31–4. doi: 10.1111/j.1440-1746.2010.06536.x 21199511

[4] Shah SC, Piazuelo MB, Kuipers EJ. AGA Clinical Practice Update on the Diagnosis and Management of Atrophic Gastritis: Expert Review. Gastroenterology. 2021;161(4):1325-1332.e7. 10.1053/j.gastro.2021.06.078 PMC8740554 34454714

[5] Fang JY, Du YQ, Liu WZ. Chinese consensus on chronic gastritis (2017, Shanghai). J Dig Dis. 2018;19(4):182–203. 29573173 10.1111/1751-2980.12593

[6] Bordin D, Livzan M. History of chronic gastritis: How our perceptions have changed. World J Gastroenterol. 2024;30(13):1851–8. doi: 10.3748/wjg.v30.i13.1851 38659477 PMC11036504

[7] Yang L, Liu X, Zhu J, Zhang X, Li Y, Chen J, et al. Progress in traditional Chinese medicine against chronic gastritis: From chronic non-atrophic gastritis to gastric precancerous lesions. Heliyon. 2023;9(6):e16764. doi: 10.1016/j.heliyon.2023.e16764 37313135 PMC10258419

[8] Lilienfeld DE, Garagliano CF, Lilienfeld AM. Letter: Model for gastric cancer epidemiology. Lancet. 1976;1(7949):45. doi: 10.1016/s0140-6736(76)92948-2 54552

[9] Wen J, Wu S, Ma X, et al. Zuojin Pill attenuates Helicobacter pylori-induced chronic atrophic gastritis in rats and improves gastric epithelial cells function in GES-1 cells. J Ethnopharmacol. 2022;285:114855. 34808298 10.1016/j.jep.2021.114855

[10] Yang T, Wang R, Liu H. Berberine regulates macrophage polarization through IL-4-STAT6 signaling pathway in Helicobacter pylori-induced chronic atrophic gastritis. Life Sci. 2021;266:118903. 33340526 10.1016/j.lfs.2020.118903

[11] Ansari S, Yamaoka Y. Helicobacter pylori Virulence Factors Exploiting Gastric Colonization and its Pathogenicity. Toxins (Basel). 2019;11(11):677. doi: 10.3390/toxins11110677 31752394 PMC6891454

[12] Waldum H, Fossmark R. Inflammation and Digestive Cancer. Int J Mol Sci. 2023;24(17):13503. 37686307 10.3390/ijms241713503 PMC10487643

[13] Qian S, Golubnitschaja O, Zhan X. Chronic inflammation: key player and biomarker-set to predict and prevent cancer development and progression based on individualized patient profiles. EPMA J. 2019;10(4):365–81. doi: 10.1007/s13167-019-00194-x 31832112 PMC6882964

[14] Bockerstett KA, Lewis SA, Noto CN, Ford EL, Saenz JB, Jackson NM, et al. Single-Cell Transcriptional Analyses Identify Lineage-Specific Epithelial Responses to Inflammation and Metaplastic Development in the Gastric Corpus. Gastroenterology. 2020;159(6):2116-2129.e4. doi: 10.1053/j.gastro.2020.08.027 32835664 PMC7725914

[15] Pan W, Liu C, Ren T, Chen X, Liang C, Wang J, et al. Exploration of lncRNA/circRNA-miRNA-mRNA network in patients with chronic atrophic gastritis in Tibetan plateau areas based on DNBSEQ-G99 RNA sequencing. Sci Rep. 2024;14(1):9212. doi: 10.1038/s41598-024-59836-4 38649401 PMC11035649

[16] Weng J, Wu X-F, Shao P, Liu X-P, Wang C-X. Medicine for chronic atrophic gastritis: a systematic review, meta- and network pharmacology analysis. Ann Med. 2023;55(2):2299352. doi: 10.1080/07853890.2023.2299352 38170849 PMC10769149

[17] Selvan TG, Gollapalli P, Kumar SHS, Ghate SD. Early diagnostic and prognostic biomarkers for gastric cancer: systems-level molecular basis of subsequent alterations in gastric mucosa from chronic atrophic gastritis to gastric cancer. J Genet Eng Biotechnol. 2023;21(1):86. doi: 10.1186/s43141-023-00539-0 37594635 PMC10439097

[18] Chen X, Zhu S, Huang C, Liu J, Wang J, Cui S. Bioinformatic analyses reveal lysosomal-associated protein transmembrane 5 as a potential therapeutic target in lipotoxicity-induced injury in diabetic kidney disease. Ren Fail. 2024;46(2):2359638. doi: 10.1080/0886022X.2024.2359638 38832484 PMC11151807

[19] Korsunsky I, Millard N, Fan J, Slowikowski K, Zhang F, Wei K, et al. Fast, sensitive and accurate integration of single-cell data with Harmony. Nat Methods. 2019;16(12):1289–96. doi: 10.1038/s41592-019-0619-0 31740819 PMC6884693

[20] Wang J, Li C, Du L. Experimental validation for mechanisms of Qizhiweitong particles against chronic non-atrophic gastritis based on metabolomics and network pharmacology. J Pharm Biomed Anal. 2023;234:115549. 37390603 10.1016/j.jpba.2023.115549

[21] Park HK, Park SH, Lee M, Kim GR, Park M, Yang SC, et al. Secretory phospholipase A2-X (Pla2g10) is a novel progesterone receptor target gene exclusively induced in uterine luminal epithelium for uterine receptivity in mice. Cell Biosci. 2020;10(1):132. doi: 10.1186/s13578-020-00495-z 33292460 PMC7678068

[22] Zhou W, Zhang H, Wang X, Kang J, Guo W, Zhou L, et al. Network pharmacology to unveil the mechanism of Moluodan in the treatment of chronic atrophic gastritis. Phytomedicine. 2022;95:153837. doi: 10.1016/j.phymed.2021.153837 34883416

[23] Zhang T, Yu W, Cheng X, Yeung J, Ahumada V, Norris PC, et al. Up-regulated PLA2G10 in cancer impairs T cell infiltration to dampen immunity. Sci Immunol. 2024;9(94):eadh2334. doi: 10.1126/sciimmunol.adh2334 38669316

[24] Ogden HL, Lai Y, Nolin JD. Secreted phospholipase A2 group X acts as an adjuvant for type 2 inflammation, leading to an allergen-specific immune response in the lung. J Immunol. 2020;204(12):3097–107. doi: 10.4049/jimmunol.2000145 32341057 PMC7375513

[25] Zhang P, Yang M, Zhang Y. Dissecting the single-cell transcriptome network underlying gastric premalignant lesions and early gastric cancer. Cell Reports. 2019;27(6):1934–47. doi: 10.1016/j.celrep.2019.04.012 31067475

[26] Massagué J, Sheppard D. TGF-β signaling in health and disease. Cell. 2023;186(19):4007–37. doi: 10.1016/j.cell.2023.07.036 37714133 PMC10772989

[27] Yuan X-N, Liu Q, Shao Y-C, Guan X-Q, Yang Z-L, Chu M-F, et al. Mettl3 synergistically regulates TGF-β/SMAD2/3 to promote proliferation and metastasis of gastric cancer. Am J Cancer Res. 2023;13(7):3185–202. 37560008 PMC10408465

[28] Jaiswal A, Rehman R, Dutta J, Singh S, Ray A, Shridhar M, et al. Cellular Distribution of Secreted Phospholipase A2 in Lungs of IPF Patients and Its Inhibition in Bleomycin-Induced Pulmonary Fibrosis in Mice. Cells. 2023;12(7):1044. doi: 10.3390/cells12071044 37048117 PMC10092981

[29] Dennis EA, Cao J, Hsu Y-H, Magrioti V, Kokotos G. Phospholipase A2 enzymes: physical structure, biological function, disease implication, chemical inhibition, and therapeutic intervention. Chem Rev. 2011;111(10):6130–85. doi: 10.1021/cr200085w 21910409 PMC3196595

[30] Yamamoto K, Hakoi H, Nomura S. The roles of sPLA2s in skin homeostasis and disease. Biomolecules. 2023;13(4):668. 37189415 10.3390/biom13040668 PMC10135803

[31] Miki Y, Taketomi Y, Kidoguchi Y, Yamamoto K, Muramatsu K, Nishito Y, et al. Group IIA secreted phospholipase A2 controls skin carcinogenesis and psoriasis by shaping the gut microbiota. JCI Insight. 2022;7(2):e152611. doi: 10.1172/jci.insight.152611 35076024 PMC8855835

[32] Murakami M, Sato H, Taketomi Y. Modulation of immunity by the secreted phospholipase A2 family. Immunol Rev. 2023;317(1):42–70. doi: 10.1111/imr.13205 37035998

[33] Ji W, Huo Y, Zhang Y, et al. Revealing therapeutic targets and mechanism of baicalin for anti-chronic gastritis using proteomic analysis of the gastric tissue. J Chromatogr B Analyt Technol Biomed Life Sci. 2022;1196:123214. 10.1016/j.jchromb.2022.123214 35303499

[34] Huang X, Zhou Y, Sun Y, Wang Q. Intestinal fatty acid binding protein: A rising therapeutic target in lipid metabolism. Prog Lipid Res. 2022;87:101178. doi: 10.1016/j.plipres.2022.101178 35780915

[35] Sekimori T, Fukunaga K, Oizumi H, Baba T, Totsune T, Takeda A, et al. FABP2 is Involved in Intestinal α-Synuclein Pathologies. J Integr Neurosci. 2024;23(2):44. doi: 10.31083/j.jin2302044 38419457

[36] Shen X, Zhang Y, Ji X, Li B, Wang Y, Huang Y, et al. Long Noncoding RNA lncRHL Regulates Hepatic VLDL Secretion by Modulating hnRNPU/BMAL1/MTTP Axis. Diabetes. 2022;71(9):1915–28. doi: 10.2337/db21-1145 35771993 PMC9862400

[37] Peng H, Chiu T-Y, Liang Y-J, Lee C-J, Liu C-S, Suen C-S, et al. PRAP1 is a novel lipid-binding protein that promotes lipid absorption by facilitating MTTP-mediated lipid transport. J Biol Chem. 2021;296:100052. doi: 10.1074/jbc.RA120.015002 33168624 PMC7949078

[38] Wang N, Huang X, Long Q. Lipid metabolic-related signature CYP19A1 is a potential biomarker for prognosis and immune cell infiltration in gastric cancer. J Inflamm Res. 2022;15:5075–88. 36091333 10.2147/JIR.S378212 PMC9462950

[39] Zubiaur P, Rodríguez-Antona C, Boone EC. PharmVar GeneFocus: CYP4F2. Clin Pharmacol Ther. 2024. 10.1002/cpt.3405 PMC11452279 39135485

[40] Cai L, Yu SZ, Zhan ZF. Cytochrome P450 2E1 genetic polymorphism and gastric cancer in Changle, Fujian Province. World J Gastroenterol. 2001;7(6):792–5. doi: 10.3748/wjg.v7.i6.792 11854903 PMC4695596

[41] Malakar M, Devi KR, Phukan RK, Kaur T, Deka M, Puia L, et al. CYP2E1 genetic polymorphism with dietary, tobacco, alcohol habits, H. pylori infection status and susceptibility to stomach cancer in Mizoram, India. Asian Pac J Cancer Prev. 2014;15(20):8815–22. doi: 10.7314/apjcp.2014.15.20.8815 25374213

