# Supplementary material for: Increased secreted PLA2 in epithelial cells promotes the progression of chronic non-atrophic gastritis to chronic atrophic gastritis through the TGF-β signaling
Source: PLoS One. 2026 Mar 4;21(3):e0343531. doi: 10.1371/journal.pone.0343531 (PMC12959716; doi:10.1371/journal.pone.0343531)
Supplement: S1 Table — (DOCX) [file pone.0343531.s001.docx]

Supplement Table 1. Nucleotide sequences of primers used for RT-PCR amplification.

| **Target gene** | | **Forward primer** | **Reverse primer** |
| --- | --- | --- | --- |
| **TNF-α**  **IL-8**  **ACE2**  **FABP2 MTTP**  **CYP450** | AGGCACTCAGATCATCTTCTC  CCCTGTCCTGCTGCTGCTG  GCCTGTTTCTGGGTTGACTG  GTCTCCACCCTGTGCTCC  CAGGCAAGTAGCAGGAGGAG  CAGCCAGCGTCTCCTCAG | | AGGTTTGCTACGACATGGTC  AGGCTTGGTGGTCCAGGACT  CAGGTGCTGAAGTGCTGAGT  CCCTTGTCAGCTTGGCTG  GCTCCAGTAGAAGGCTGAGG  GGTCTTTGCCATGACGGT |
