# Supplementary material for: Increased secreted PLA2 in epithelial cells promotes the progression of chronic non-atrophic gastritis to chronic atrophic gastritis through the TGF-β signaling
Source: PLoS One. 2026 Mar 4;21(3):e0343531. doi: 10.1371/journal.pone.0343531 (PMC12959716; doi:10.1371/journal.pone.0343531)
Supplement: S2 Table — (DOCX) [file pone.0343531.s002.docx]

Supplement Table 2. Rat body weight at different time points

| The modeling phase | body weight (g): ^**^*P*<0.01 vs. the Normal group | | | |
| --- | --- | --- | --- | --- |
|  | **Normal group** | **CNAG group** | **PLA2G10 siRNA group** | **TGF-β siRNA group** |
| Day 0 （2-month-old ） | 203.82±14.32 | 205.92±17.13 | 201.34±11.73 | 203.67±13.26 |
| Day 10 | 239.34±21.58 | 227.36±13.45^**^ | 225.32±14.84^**^ | 223.40±19.65^**^ |
| Day 20 | 262.57±17.63 | 242.17±14.96^**^ | 245.72±10.41^**^ | 244.98±16.05^**^ |
| Day 30 | 298.83±14.28 | 264.96±12.03^**^ | 268.39±23.57^**^ | 263.44±20.92^**^ |
| Day 40 | 335.46±22.91 | 288.42±17.15^**^ | 291.66±14.13^**^ | 292.71±11.63^**^ |
| Day 50 | 357.92±19.46 | 305.79±23.12^**^ | 303.19±17.94^**^ | 303.26±18.07^**^ |
| Day 60 | 382.11±10.32 | 322.25±20.67^**^ | 324.94±20.29^**^ | 325.59±13.95^**^ |
| Day 70 | 408.68±12.57 | 347.64±11.89^**^ | 348.51±11.22^**^ | 343.82±17.26^**^ |
| Day 80 | 424.35±20.11 | 363.31±15.24^**^ | 367.28±18.66^**^ | 368.37±20.45^**^ |
| The intervention phase | ^**^*P*<0.01 vs. the Normal group; ^##^*P*<0.01 vs. the CNAG group | | | |
| Day 90 （5-month-old ） | 445.74±15.90 | 377.90±18.33^**^ | 378.76±15.73^**^ | 375.69±13.58^**^ |
| Day 97 | 459.29±11.46 | 392.58±21.95^**^ | 395.34±13.41 | 398.12±10.63 |
| Day 104 | 471.61±18.79 | 405.12±13.67^**^ | 417.63±22.76^##^ | 422.78±18.48^##^ |
| Day 111 | 484.88±13.02 | 419.85±16.71^**^ | 431.87±19.57^##^ | 438.24±21.79^##^ |
| Day 118 （7-month-old ） | 499.53±16.48 | 438.47±12.88^**^ | 452.15±10.98^##^ | 456.27±18.22^##^ |
